# A Retrofit Theory to Prevent Fatigue Crack Initiation in Aging Riveted Bridges Using Carbon Fiber-Reinforced Polymer Materials

**DOI:** 10.3390/polym8080308

**Published:** 2016-08-18

**Authors:** Elyas Ghafoori, Masoud Motavalli

**Affiliations:** 1 Empa, Swiss Federal Laboratories for Materials Science and Technology, Structural Engineering Research Laboratory, Überlandstrasse 129, Dübendorf 8600, Switzerland; masoud.motavalli@empa.ch; 2 School of Civil Engineering, University of Tehran, Tehran 1417466191, Iran

**Keywords:** carbon fiber-reinforced polymer, bonded, unbonded, high-cycle fatigue life, prevention of crack initiation, rehabilitation, old steel members, riveted metallic bridges, constant life diagram, strengthening

## Abstract

Most research on fatigue strengthening of steel has focused on carbon fiber-reinforced polymer (CFRP) strengthening of steel members with existing cracks. However, in many practical cases, aging steel members do not yet have existing cracks but rather are nearing the end of their designed fatigue life. Therefore, there is a need to develop a “proactive” retrofit solution that can prevent fatigue crack initiation in aging bridge members. Such a proactive retrofit approach can be applied to bridge members that have been identified to be deficient, based on structural standards, to enhance their safety margins by extending the design service life. This paper explains a proactive retrofit design approach based on constant life diagram (CLD) methodology. The CLD approach is a method that can take into account the combined effect of alternating and mean stress magnitudes to predict the high-cycle fatigue life of a material. To validate the retrofit model, a series of new fatigue tests on steel I-beams retrofitted by the non-prestressed un-bonded CFRP plates have been conducted. Furthermore, this paper attempts to provide a better understanding of the behavior of un-bonded retrofit (UR) and bonded retrofit (BR) systems. Retrofitting the steel beams using the UR system took less than half of the time that was needed for strengthening with the BR system. The results show that the non-prestressed un-bonded ultra-high modulus (UHM) CFRP plates can be effective in preventing fatigue crack initiation in steel members.

## 1. Introduction

### 1.1. State of the Art

The conventional method of repairing or strengthening aging steel structures often involves bulky and heavy plates that are difficult to fix and are prone to corrosion of their own. Carbon fiber-reinforced polymer (CFRP) has great potential to enhance steel structures in terms of increased strength, ductility, energy absorption and fatigue life [1,2,3,4,5,6,7,8,9,10,11,12,13,14,15,16,17,18,19,20]. Tavakkolizadeh and Saadatmanesh [18] investigated the performance of notched steel beams retrofitted with CFRP patches under medium cycle fatigue loading. The test results for a four-point bending test scheme with a cyclic loading frequency between 5 and 10 Hz showed that the CFRP patch extended the fatigue life more than threefold. Rizkalla et al. [14] conducted fatigue tests on CFRP-strengthened steel-concrete composite beams. The two strengthened beams showed superior performance compared with the un-strengthened reference beam. The test results indicated that the different bonding techniques used did not substantially affect the fatigue behavior of the strengthening system. Täljsten et al. [21] conducted several laboratory tests to improve the fatigue life of old steel plates with a center notch using bonded CFRP laminates. Using non-prestressed CFRP plates can increase the fatigue life nearly fourfold compared with the unstrengthened reference specimen. A similar experimental study was conducted by Huawen et al. [22] on steel plates strengthened with pre-stressed CFRP laminates subjected to cyclic tension loadings. The pre-cracked and subsequently strengthened specimens again showed that an increased pre-stressing force increased the fatigue life of the retrofitted members. Ghafoori and Motavalli [23] presented an analytical model to calculate the stress intensity factor of cracked steel I-beams under flexural or axial loading. Aljabar et al. [24] studied the effect of crack orientation on the fatigue behavior of the retrofit steel plates.

Ghafoori et al. [25] developed a pre-stressed un-bonded retrofit (PUR) system to avoid the drawbacks of the bonded joints. The performance of notched steel beams strengthened with the PUR system was compared with that of notched steel beams strengthened with the traditional pre-stressed bonded retrofit (PBR) system. Both systems generally showed a good fatigue behavior. Specimens strengthened with the PBR system caused a local stress concentration in the CFRP laminate under the cracked beam section that resulted in a slower crack growth rate, whereas the induced strain was evenly distributed over the laminate length in the PUR system. For notched steel beams retrofitted with non-prestressed and prestressed bonded CFRP laminates, Ghafoori et al. [26] proposed a theoretical model based on linear elastic fracture mechanics (LEFM) to estimate the required pre-stressing level to completely arrest crack propagation. Laboratory tests with digital image correlation (DIC) measurements were conducted to validate the LEFM model. A series of experiments have been performed to compare the flexural performance of metallic beams strengthened by bonded and un-bonded CFRP laminates with Young’s moduli ranging from 140 to 440 GPa [27,28]. Furthermore, a series of fatigue tests were performed on steel beams retrofitted with the bonded CFRP plates with different Young’s moduli [29,30].

A trapezoidal PUR (TPUR) system was recently developed and tested in the laboratory for static loading [31]. Ghafoori et al. [32] studied the optimum level of CFRP pre-stress required for fatigue strengthening of riveted metallic girders. The TPUR system was then used for fatigue strengthening of riveted girders in a 120-year-old historic railway bridge in Switzerland [33]. A finite element (FE) model was created to determine the most fatigue-prone details of the bridge [34]. The TPUR system was then installed on the bridge girders. The long-term behavior of the retrofitted girders and the pre-stressing system have since been monitored using a wireless sensor network (WSN) system [33].

### 1.2. Motivation

As can be observed from the last section, there have been several studies on fatigue strengthening of metallic members using CFRP materials (e.g., [19,20,21,25,26,35]). Nevertheless, the majority of these studies have used the CFRP materials for fatigue strengthening of members with existing cracks. In this type of study, CFRP material is used to decrease the overall stress intensity factor (SIF) at the crack tip to reduce the fatigue crack growth (FCG) rate. The application of such a strengthening method is limited to cases in which cracks have already been detected in existing elements. This type of research represents a “reactive” strengthening approach and suggests a solution only for after crack initiation; however, it does not make any retrofit recommendation to prevent fatigue crack initiation. Nonetheless, municipalities often look for strengthening solutions that can prevent fatigue crack initiation rather than late strengthening after crack initiation. This is because the strengthening of members with existing cracks generally involves substantially more work and monitoring. There is a clear need to develop “proactive” strengthening approaches to avoid any fatigue crack initiation in aging metallic members that are nearing their designed fatigue lives. Such proactive strengthening methods may be applied to elements of existing bridges that have been identified to be deficient according to existing standards in attempts to increase their safety shoulders by extending the design service life of the metallic members.

One of the primary aims of this paper is to explain a proactive design approach based on constant life diagram (CLD) methodology for fatigue strengthening of metallic members. The proposed design approach is capable of taking into account the combined effect of alternating and mean stress magnitudes. The results of a new series of static and fatigue experiments on beams strengthened with the un-bonded CFRP plates will be presented in this paper.

Furthermore, this paper attempts to provide a better understanding of the behavior of bonded and un-bonded CFRP-retrofit systems. Unlike bonded retrofit (BR) systems, un-bonded retrofit (UR) systems work without any glue and use a pair of mechanical clamps, which work based only on friction. The UR systems have several advantages over the BR systems: they can be applied to rough (e.g., corroded) or obstructed (e.g., riveted) surfaces, and they offer rapid installation (i.e., no surface preparation prior to bonding and glue application is necessary). The UR systems are particularly good for strengthening heritage bridges, where the reversibility of the retrofitted elements to the original unstrengthened form is important. Unlike the BR systems, the UR systems offer an adjustable pre-stress level and can be easily detached from the structure by disassembling the mechanical clamps.

This paper presents, for the first time, the results of a new experimental program on fatigue strengthening of steel I-beams using non-prestressed un-bonded CFRP plates. The main goal of the paper is to show that non-prestressed un-bonded CFRP plates can prevent fatigue crack initiation in steel members, provided that high or ultra-high modulus CFRP plates are used.

## 2. Fatigue Theories

Low-cycle and high-cycle fatigue are the two most common types of fatigue theories. The former is used when the number of cycles to failure (*N*) is less than or equal to 10^3^, whereas the latter is utilized for *N* > 10^3^. Due to the long service life of civil-type infrastructure, high-cycle fatigue is usually most relevant. In the following, the stress-life method, which is applicable for high-cycle fatigue [36], is described. The cyclic loadings usually show a sinusoidal pattern with a minimum (σ_min_) and a maximum stress (σ_max_) or, alternatively, can be defined by the mean stress (σ_m_) and the stress amplitude (σ_a_) as given by

(1) $$\sigma_{m}=\frac{\sigma_{\max}+\sigma_{\min}}{2}$$

(2) $$\sigma_{a}=\left|\frac{\sigma_{\max}-\sigma_{\min}}{2}\right|$$

Using these two characteristic values, the stress ratio (*R*) is defined as:

(3) $$R=\frac{\sigma_{\min}}{\sigma_{\max}}$$

**Figure 1:**
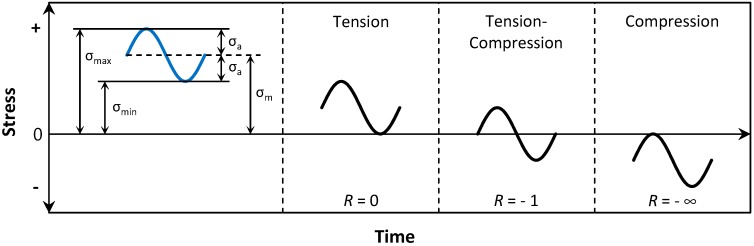
Stress cycles with different stress ratios of *R* = 0, −1 and −∞.

Figure 1 illustrates these definitions along with three sample stress ratios of *R* = 0, −1 and −∞.

### 2.1. Miner Linear Damage Accumulation

To investigate the influence of a certain load pattern on the total cumulative fatigue damage, *D*, the Miner linear damage accumulation rule can be considered:

(4) $$D=\sum \frac{n_{i}}{N_{i}}\leq 1$$

where *N*_i_ and *n*_i_ are the number of cycles to failure and the applied number of cycles both at stress level σ_i_, respectively. Until the overall accumulated damage remains below unity, fatigue failure does not happen.

A stress level *σ*_k_, which does not lead to a fatigue failure within two million load cycles, is assumed to feature an infinite number of cycles to failure. Therefore, its contribution to the cumulative damage is negligible, and

(5) $$\underset{N_{k}\to \infty }{\lim}\frac{n_{k}}{N_{k}}=0$$

### 2.2. Stress Concentration at Notches

**Figure 2:**
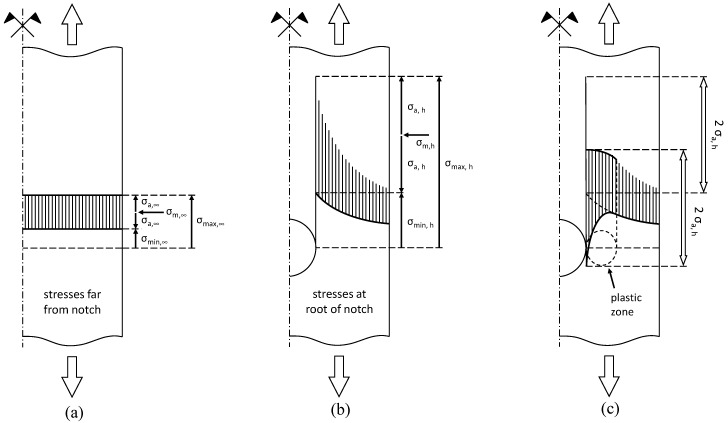
Stresses at (**a**) gross cross-section; (**b**) at notch in elastic and (**c**) plastic range.

Figure 2 depicts the stress distribution of a notched plate under cyclic tension (*R* > 0). The cross-section in Figure 2a, which is relatively far away from the notch and is denoted by the subscript ∞, exhibits uniformly distributed stresses. At the hole cross-section, which is denoted by subscript h, the hole reduces the cross-sectional area and therefore generally increases the stresses in the remaining net section. Furthermore, the hole leads to a stress concentration. With moderate loading, the stresses remain in the elastic region (see Figure 2b). However, if the load increases, the peak stress exceeds the yield point and forms a plastic zone at the hole edge in the first load cycle (see Figure 2c). The yielding decreases both the maximum and minimum stress with the same magnitude (and may even turn to compression) at the hole while the applied external load remains in tension *R* > 0. Consequently, the mean stress decreases by the same magnitude, which may have a beneficial influence on the fatigue behavior of the specimen.

Due to the size of measurement devices (e.g., strain gauge), it is difficult to directly measure the above stress concentrations. Alternatively, far-field stresses can be measured at some distance away from the hole where there is a constant stress distribution (see Figure 2a). Using the following formulation, which includes the width of the plate (*w*) and the diameter of the hole (*d*), the far-field stresses can be translated to the peak stresses at the notch [32]:

(6) $$\sigma_{h}=k_{f}\frac{w}{w-d}\sigma_{\infty }=k_{tot}\cdot \sigma_{\infty }$$

The fatigue stress concentration factor (*k*_f_) depends on the material properties and the geometry of the member and is given

(7) $$k_{f}=1+q\left(k_{t}-1\right)$$

Neuber proposed the following relationship as the notch sensitivity parameter (*q*), which is normally between zero and unity

(8) $$q=\frac{1}{1+\frac{\sqrt{a}}{\sqrt{r}}}$$

where *r* is the root notch radius. Furthermore, $\sqrt{a}$ is the Neuber constant for steels and wrought irons, which is given as a function of the ultimate tensile stress (*S*_ut_), by [36]

(9) $$\sqrt{a}=\frac{147}{S_{ut}}$$

The geometric stress concentration factor (*k*_t_) is usually derived from experiments and can be read from design diagrams [33]. Note that if the theoretical peak stresses at the notch derived by the described method exceed the yield strength of the material, a non-linear stress analysis must be conducted to determine the stress distribution around the notch after the first load cycle. Such an analysis can account for stress redistribution during yielding and strain hardening effects and may be performed using a finite element (FE) package.

### 2.3. Constant Life Diagram (CLD) Approach

**Figure 3:**
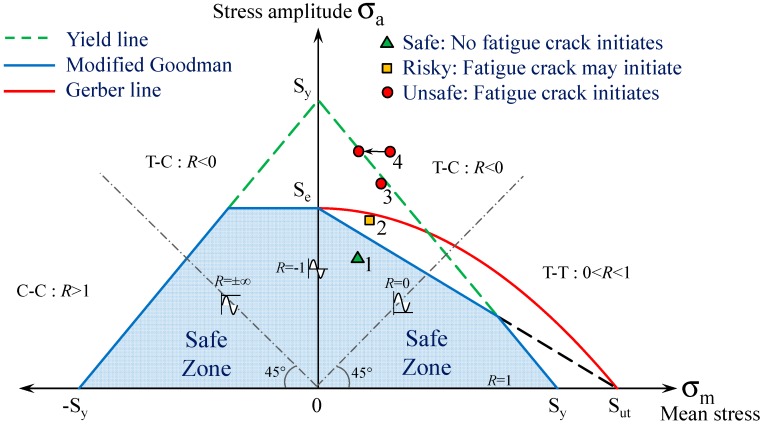
Illustration of different fatigue failure zones in the constant life diagram (CLD) approach.

Figure 3 shows a CLD that is built based on the yield stress (*S*_y_), the fatigue endurance limit (*S*_e_) and *S*_ut_ of the material. The endurance limits are usually determined for the stress ratio of *R* = −1 (see Figure 1). For non-zero mean stresses, the fatigue endurance limit must be calculated using models developed based on laboratory tests (e.g., [37]). Goodman proposed a straight line between the known points [*S*_ut_;0] and [0;*S*_e_], whereas Gerber suggested a parabola through the same two points. Both of the models are applicable for ductile metals. The Goodman line is characterized by

(10) $$\frac{\sigma_{a}}{S_{e}}+\frac{\sigma_{m}}{S_{ut}}=1$$

The criterion equation for the Gerber line is

(11) $$\frac{\sigma_{a}}{S_{e}}+{\left(\frac{\sigma_{m}}{S_{ut}}\right)}^{2}=1$$

Furthermore, the yield strength is shown with two green dashed lines and is defined as

(12) $$\frac{\sigma_{a}}{S_{y}}+\frac{\sigma_{m}}{S_{y}}=1\;0\leq \sigma_{m}\leq S_{y}$$

(13) $$\frac{\sigma_{a}}{S_{y}}-\frac{\sigma_{m}}{S_{y}}=1\;-S_{y}\leq \sigma_{m}\leq 0$$

In Figure 3, the blue lines represent an envelope that includes all cyclic loads with an infinite life. The Goodman line, which is part of the envelope, is conservative and therefore usually used for design purposes. The Gerber criterion is more realistic but can be non-conservative due to the high scatter of fatigue failures. Therefore, a structural detail that is subjected to a cyclic stress represented by “Point 1” will exhibit an infinite fatigue life. If the cyclic load is increased to “Point 2”, which is between the Gerber and the Goodman line, fatigue crack will occur with a probability of less than 50%. The risk of failure increases to 50% as the load point approaches the non-conservative Gerber criterion. “Point 3” lies outside of the Gerber criterion. A member characterized by this point shows limited fatigue life (with a probability more than 50%) and fails after a certain number of load cycles. The CLD itself gives no information about the number of cyclic loads prior to failure; however, this can be calculated using the cumulative damage method if needed. “Point 4” sits outside the yielding line and therefore only corresponds to a theoretical initial position. Due to local yielding at the stress concentration (see Figure 2c) at the first load cycle, the mean stress level decreases, as previously explained. In the CLD, this process leads to a horizontal shift onto the yielding line. Because “Point 4” remains outside the safe zone also after yielding, an infinite fatigue life cannot be reached.

## 3. Strengthening Theory

**Figure 4:**
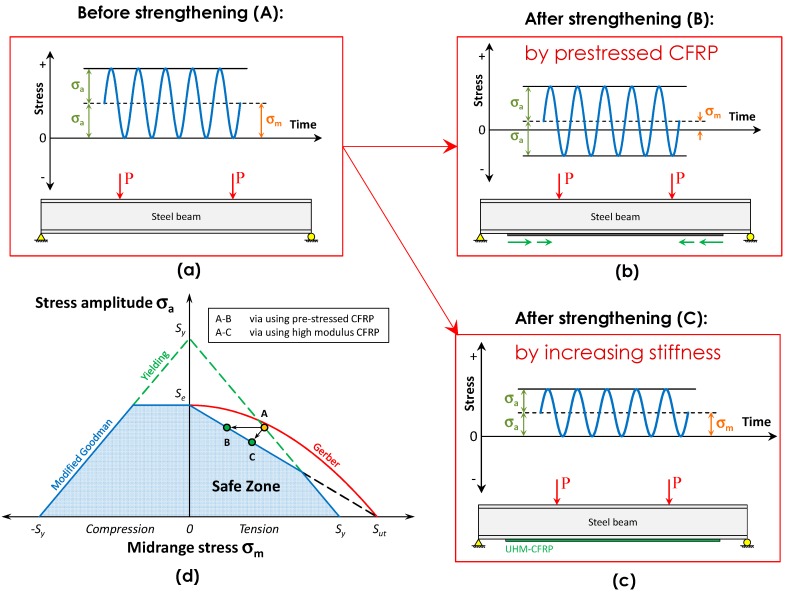
The stress at the bottom flange of a beam subjected to external cyclic loading, P, when the beam is (**a**) not strengthened; (**b**) strengthened with the pre-stressed NM CFRP plate or (**c**) strengthened with the ultra-high modulus (UHM) plates; (**d**) A CLD that indicates the stresses prior to and after strengthening.

Consider a beam subjected to external cyclic loading, P, in a four-point bending test set-up, as shown in Figure 4a. The time-history of the stress at the beam bottom flange prior to strengthening is shown in Figure 4a. Assume that the applied cyclic load refers to Point A in the CLD shown in Figure 4d, which is in the unsafe zone and indicates a finite fatigue life. Two approaches for fatigue retrofitting of steel members are presented in this section.

In the first approach, the beam is retrofitted using pre-stressed normal modulus (NM) CFRP plates. Therefore, the mean stress is substantially reduced; however, the stress amplitude decreases only negligibly. Note that Point B is in the safe zone and the metallic member will have an infinite fatigue life after strengthening. Hence, path A–B in Figure 4d indicates the effect of the pre-stressed NM CFRP plates.

The second approach of strengthening a deficient steel member is to decrease the alternating stress by substantially increasing the stiffness of the steel member. The stiffness increase can be achieved by retrofitting with non-prestressed ultra-high modulus (UHM) CFRP plates. Therefore, the stress amplitude and the mean stress will be reduced by the same factor, as shown in Figure 4c. In Figure 4d, path A–C exhibits a stiffening effect. Point C is in the safe zone, and the steel member will have an infinite fatigue life under the applied cyclic loads after strengthening. Note that the stiffening of a steel member can be performed using several methods, such as by increasing the thickness, the width or the Young’s modulus of the CFRP plate. In this paper, only increasing the CFRP Young’s modulus is discussed.

Note that if the beam is retrofitted using a pre-stressed CFRP UHM plate, a combined effect of decreasing both the mean and alternating stresses will be achieved, and the strengthening path will sit between paths A–B and A–C. However, pre-stressing the UHM plate is not recommended, as UHM plates are often brittle and have relatively lower tensile strength than NM plates.

## 4. Prediction Behavior of Retrofitted Beams

The last section showed that it is possible to use pre-stressed or stiff CFRP plates to increase the fatigue life of a metallic member. However, there remains an open question about the minimum required CFRP pre-stress level and stiffness that are required.

### 4.1. Metallic Beams Retrofitted with Bonded Carbon Fiber-Reinforced Polymer (CFRP) Plates

The authors [9,10] previously developed closed-form solutions for the flexural and the interfacial behavior of steel beams strengthened by prestressed bonded plates. The influence of various parameters such as the CFRP pre-stress level, steel grade and mechanical and geometric properties of the CFRP material on both the interfacial shear stress and the flexural behavior of the plated beam have been discussed. This paper has shown that while the mechanical and geometric properties of the plate affect the yield load capacity and the stiffness of the plated beam, the CFRP pre-stress level results not in an increased stiffness but rather an increase in the yield load capacity of the retrofitted beam. Although the magnitude of the CFRP pre-stress does not influence the stiffness of the retrofitted beam in the elastic domain, it substantially decreases the tension stresses in the beam bottom flange. In this manner, the mean stress is decreased while the alternating stress is maintained, which is in favor of the first retrofitting approach presented in Section 3. It is possible to substitute the analytical formulations of the stresses in the beam bottom flange in the Goodman criterion (i.e., Equation (10)) and calculate the minimum pre-stress level that is required to shift the detail stress from the “unsafe” to “safe” stress region, as shown in path A–B in Figure 4d. The model can also be used to determine the Young’s modulus and dimensions of the CFRP plates such that the metallic detail is shifted from a “finite-life” regime to the “infinite-life” regime, as shown in path A–C in Figure 4d. The authors performed a series of fatigue experimental studies to verify the accuracy of the methodology [30]. More details regarding the development of the analytical solutions and fatigue experimental program can be found [30].

### 4.2. Beams Retrofitted with Un-Bonded CFRP Plates

Kianmofrad et al. [38] presented techniques for using pre-stressed un-bonded retrofit systems. The un-bonded retrofit system can be used to strengthen metallic members with rough (e.g., corroded) or obstructed (e.g., riveted or bolted) surfaces. The system offers a rapid application procedure because there is no need for surface preparation prior to bond application. Kianmofrad et al. [38] introduced four variants of the prestressed un-bonded retrofit (PUR) systems: trapezoidal PUR (TPUR), triangular PUR (TriPUR), Flat PUR (FPUR), and Contact PUR (CPUR) systems. The CFRP plates in the FPUR and CPUR systems are very close to the beam bottom flange; therefore, these two systems are appropriate for retrofitting girders where there is not substantial space available beneath the bridge (e.g., because of ongoing traffic). A series of analytical solutions based on the flexibility approach has been developed [38] to predict the behavior of the metallic beams retrofitted with the PUR systems.

The results of analytical, numerical and experimental studies [38] showed that the efficiency of the PUR system is primarily dependent on the CFRP pre-stress level rather than the type of PUR system. Therefore, any variant of the PUR system that can ease application in the field can be considered for the retrofit solution. Details are available regarding the development of the analytical solutions for the four presented PUR systems [38].

Similar to the retrofit system with bonded CFRP plates, the analytical solutions provided by Kianmofrad et al. [38] can be used to predict the stresses in the bottom flange of the beam after strengthening. In this manner, the stress in the beam bottom flange after strengthening should be substituted in the Goodman criterion in Equation (10). In this paper, the results of a new series of static and fatigue experiments on steel beams retrofitted with the CPUR system will be presented. Furthermore, the results will be compared with those obtained from the specimens retrofitted using the PBR system. A critical comparison on the efficiency of these two retrofit approaches will also be conducted.

## 5. Test Set-up, Specimens and Materials

### 5.1. Test Set-up

**Figure 5:**
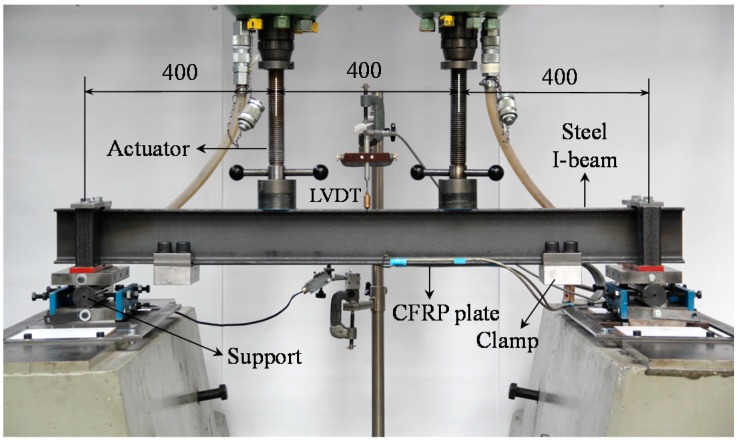
Four-point bending test set-up (dimensions in mm).

All static and fatigue tests were conducted using a four-point bending test set-up, as shown in Figure 5. Note that the LVDT is a linear variable differential transformer. The test specimens were loaded using two hydraulic jacks, each with 100 kN static and 50 kN dynamic capacity, suspended from a relatively rigid reaction frame. The beam span was 1200 mm, and the jacks produced a constant moment along a length of 400 mm in the middle of the beam.

### 5.2. Strengthening Technique

**Figure 6:**
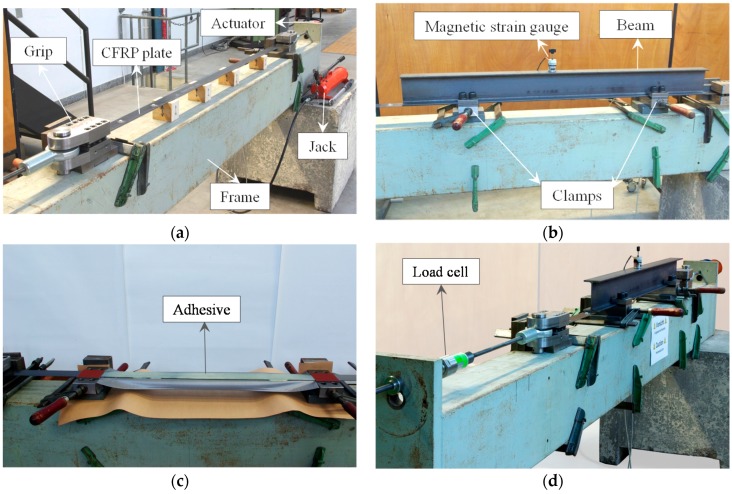
Strengthening set-up. (**a**) A manual hydraulic jack is used to stretch the CFRP plate; (**b**) mechanical clamps are used to fix the plate to the beam in the un-bonded systems; (**c**) in bonded systems, adhesive are applied on top of the CFRP plate and (**d**) the beam is placed on top of the plate for at least 24 h for curing.

Figure 6a shows a set-up that was used to prepare the samples. Two end grips holding the CFRP plate were connected through tensile bars to a reaction beam. At one end, a hydraulic actuator operated by a manual jack was used to stretch the CFRP plate. The force in the CFRP plate was measured using a load cell. Once the CFRP plate is straight, mechanical clamps were used to attach the CFRP plate to the steel beam, as shown in Figure 6b. For beams retrofitted with bonded systems, two-component adhesive was applied on the top of the pre-stressed CFRP plates (see Figure 6c). The steel beam was then placed on the top of the CFRP plate for at least 24 h (see Figure 6d). To prevent premature interfacial debonding at the ends of the plate, mechanical clamps were also used for the bonded systems. After the adhesive was cured, the CFRP plate was cut and transported to the four-point test set-up.

**Figure 7:**
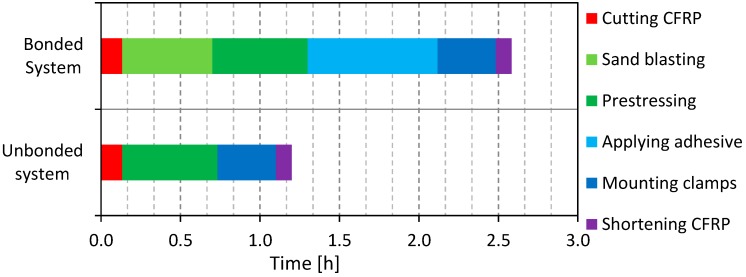
Comparison between the times needed for strengthening steel beams using the bonded retrofit (BR) and un-bonded retrofit (UR) systems.

The time required to strengthen the steel beams using the bonded and the un-bonded retrofitted systems were registered for all specimens. Figure 7 shows the average time spent for the BR and UR systems. The specimens retrofitted with the BR systems required nearly twice the duration that was needed for the UR systems. This is because the UR specimens do not need surface preparation and glue application prior to the application of the CFRP plate.

### 5.3. Specimens

**Figure 8:**
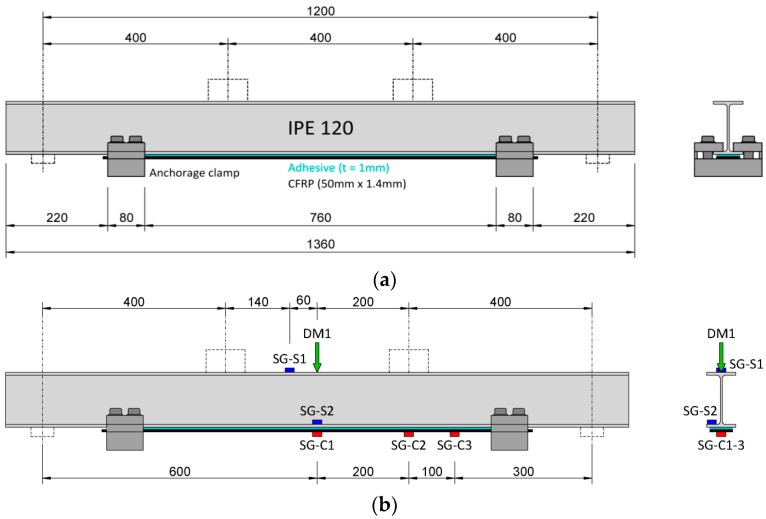

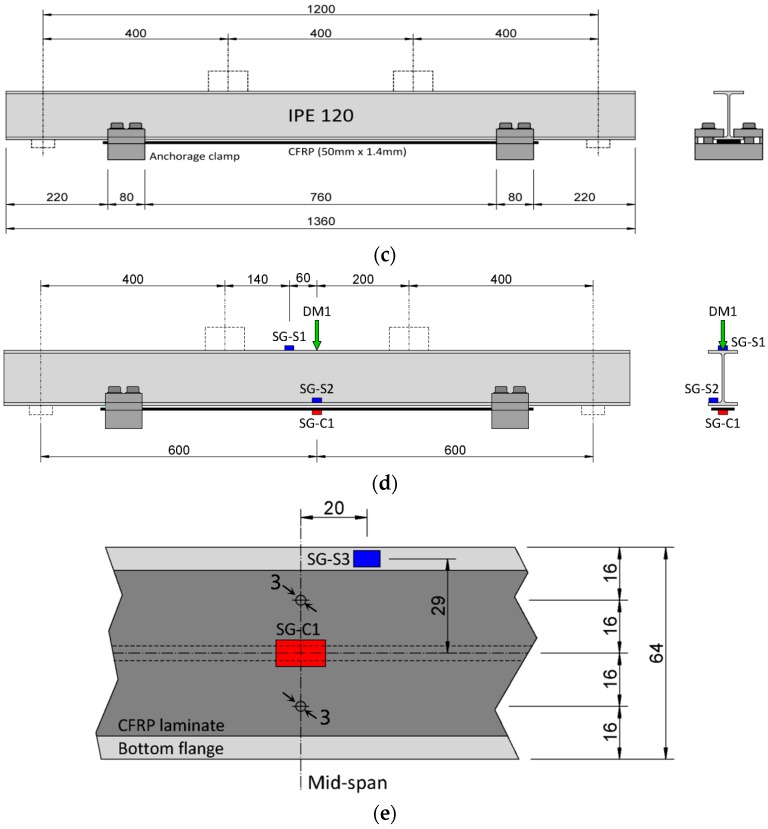
(**a**) Specimens strengthened with the BR system; (**b**) Measurement layout for the BR system; (**c**) specimens strengthened with the UR system; (**d**) measurement layout for the UR system [27]; (**e**) details of the two drilled holes in the bottom flange of the fatigue specimens, the CFRP laminate and strain gauges (dimensions in mm).

The steel profiles of IPE 120 were strengthened with the CFRP plate using the BR and UR systems. Figure 8 show the layout of the geometry, the dimensions and measurement devices of the specimens strengthened using the BR and the UR systems, respectively. To induce a stress concentration for crack initiation, two holes with diameters of 3 mm were drilled through the bottom flange at mid-span of all fatigue test specimens, as shown in Figure 8e. Figure 8e shows a schematic view of the beam bottom flange with the measurement layout and the CFRP laminate.

To monitor the strain along the CFRP plate, bonded electrical strain gauges of type 1-LY66-6/120 provided by Hottinger Baldwin Messtechnik GmbH were glued on the plates (see SG-C1, SG-C2 and SG-C3 in Figure 8b for the BR system and SG-C1 for the UR system). The strain gauges had a *k*-factor of 2.02% ± 1.0% and an electric resistance of 120 Ω ± 0.3%. Furthermore, for the static tests, magnetic strain gauges (see Figure 6b) of type FGMH-1 from Tokyo Sokki Kenkyujo Co. Ltd. (Tokyo, Japan) were used to measure the strain on the steel beams. The magnetic strain gauges (see SG-S1 and SG-S2 in Figure 8b,d) had a *k*-factor of 2.02% ± 2.0% and an electric resistance of 120 Ω ± 0.5%. During the static and fatigue tests, the mid-span (see DM1 in Figure 8b,d) deflection of the beams were measured using a LVDT.

### 5.4. Materials

The steel profiles of IPE 120 were provided by Briner AG, Winterthur, Switzerland. The steel grade was S235JR in accordance with EN 10025. Three tensile tests were conducted using tension specimens according to DIN 50125-E Standard. The results of the tensile tests are summarized in Table 1.

**Figure 9:**
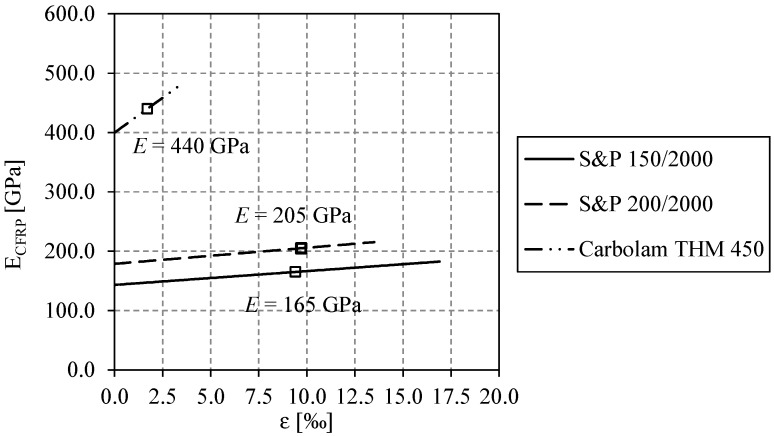
Measured Young’s modulus of the CFRP plates.

Three types of CFRP plates with different Young’s modulus were used. The NM and the high modulus (HM) were provided by the S&P Clever Reinforcement Company, Seewen, Switzerland, and were of type “S&P 150/2000” and “S&P 200/2000”, respectively. The UHM plates were provided by the Epsilon Composite, Gaillan en Médoc, France, and were of type “Carbolam THM 450”. The geometrical properties of the CFRP plates are shown in Table 2. Table 3 shows the mechanical properties of the CFRP plates specified in the data sheets from the manufacturing companies. In addition to manufacturer’s data sheets, the Young’s modulus of the various CFRP plates was previously measured using the pre-stressing set-up and an extensometer [27,28]. The results of these preliminary tests showed that the Young’s modulus increases linearly with increasing stress, as shown in Figure 9. None of the tested CFRP plates reached its specified minimal Young’s modulus (marked in Figure 9) at low stress ranges. It instead takes higher stresses (55%, 73% and 63% of their minimal tensile strength or ultimate strain for the NM CFRP, the HM CFRP and the UHM CFRP, respectively) to achieve the requested values. The observed behavior of an increasing Young’s modulus is a typical case and is due to the stiffening effects of the carbon fiber itself [39].

To bond the CFRP plate to the bottom flange of the steel profile, Araldite^®^ AW 106 with Hardener HV 953 U, produced by Huntsman, Los Angeles, CA, USA, was used. This type of adhesive has a non-linear behavior with a relatively low elastic modulus but a large strain capacity that leads to a higher interfacial fracture energy than linear adhesives with similar or even higher tensile strength [40,41]. Table 4 shows the mechanical properties of adhesive at 23 °C according to ISO 527 based on the manufacturer’s data sheet.

## 6. Test Plan and Results

### 6.1. Test Plan

The test set-up and dimensions of the specimens are similar to those presented in Section 5.2. All beams were tested using the symmetric four-point bending set-up shown in Figure 5. Seven test specimens were prepared to investigate the behavior of steel beams strengthened with non-prestressed CFRP plates (see Table 5). First, an unstrengthened reference beam B0 was tested. For each CFRP type, one BR and one UR system was tested. Prior to fatigue tests, all specimens were subjected to a static load in their linear elastic domain. The goal of such initial static loading was to examine the elastic flexural behavior of the specimens and compare the test results with those obtained from the existing analytical solutions.

An LVDT was used to measure the vertical displacement at the beam mid-span. A Pulsator-P960 hydraulic testing machine with a force control system was used to conduct the fatigue loads. To ensure no damage at the surface of the holes, the Eddy Current testing system (Elotest-B1) was used to inspect the specimens prior to fatigue tests. This non-destructive testing (NDT) system works based on the electromagnetic induction of circular coils and is commonly used to detect flaws down to 500 microns.

### 6.2. Static Test Results

Laboratory static results (in linear elastic domain) for bonded and un-bonded retrofit systems are quite beneficial for interpreting the fatigue behavior of the member after strengthening. The results of the static tests are in the linear elastic domain. Such results are very helpful for fatigue analysis as the service loads on bridges are often relatively low and the bridge elements are in the linear elastic domain. Therefore, the results of laboratory static tests can provide a better understanding of the behavior of the metallic member after strengthening subjected to service loads. In particular, such static tests can provide useful information about the stress distribution along the CFRP laminates and critical metallic detail. The results can be used for fatigue analysis to determine the lifetime of the metallic member after strengthening. Furthermore, the present paper gives an estimation of the time that is required for strengthening of the bonded and the un-bonded retrofit systems. This information will help engineers to consider different aspects of a retrofitting plan, particularly costs (e.g., required strengthening time) versus performance (e.g., post strengthening behavior).

**Figure 10:**
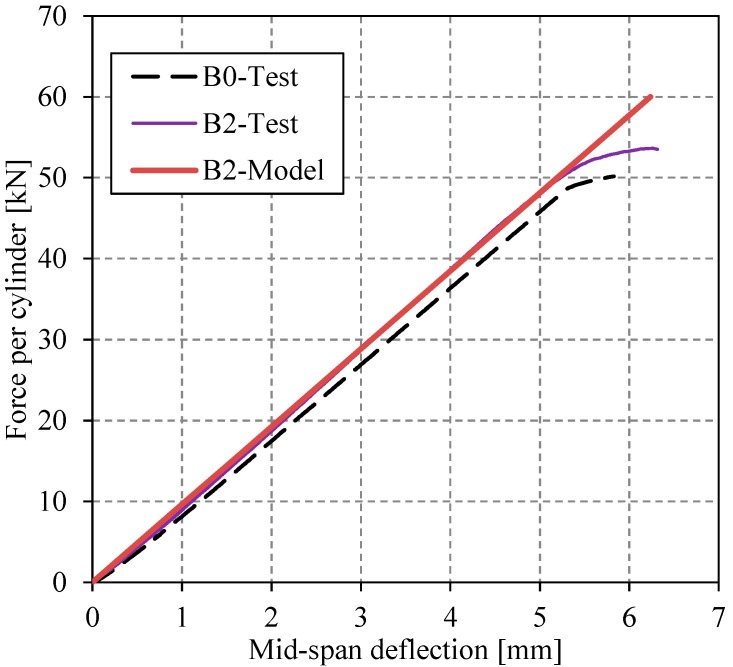
The load-deflection behavior of reference specimen B0 and specimen B2 (retrofitted with the un-bonded NM CFRP plate).

Figure 10 shows the load-deflection behaviors of reference specimens B0 and B2, which were retrofitted by the un-bonded NM CFRP plate. The behavior of the unstrengthened reference beam B0 is indicated by the black dashed line. The test results are compared with those obtained from the analytical model presented in [38], which shows a good agreement (in the linear elastic domain). Details about the analytical model can be found in [38].

**Figure 11:**
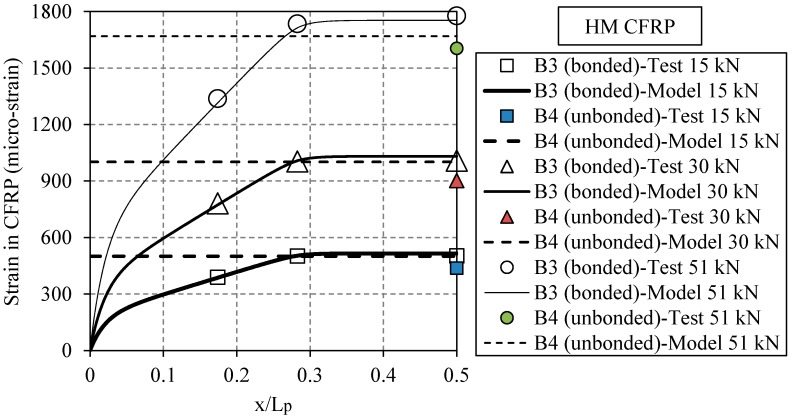
The measured and calculated strains along the CFRP plates for the specimens strengthened with bonded and un-bonded HM CFRP plates (i.e., B3 and B4, respectively).

Figure 11 compares the measured and the calculated strains along the CFRP plates versus the normalized distance from the plate end, *x*/*L*_p_, for the beams strengthened with bonded and un-bonded HM CFRP plates (i.e., B3 and B4, respectively). Elastic load levels were chosen because the proposed strengthening solution is against fatigue cracks under service loads, so load levels are relatively low and often remain in the elastic domain. The results for different actuator load levels of 15, 30 and 51 kN in the elastic domain are shown. For each load level, the behavior of bonded and un-bonded retrofitted specimens is compared. Note that the units for “micro-strain” refers to μm/m. In this figure, the markers show the experimental results as read from the applied strain gauges, and the lines show the analytical predications for bonded [30] and un-bonded [38] systems. Due to the existing symmetry, the results of only one side of the CFRP plates are shown. Note that for the un-bonded plates, the strain is constant along the CFRP plate. Note that in Figure 11, the strain at the beginning of the plate is zero and then increases when approaches the beam mid-span. The shape of the theoretical graphs (with solid lines) represents three distinct zones. The strain at the beginning of the plate (i.e., *x*/*L*_p_ = 0) is zero and then increases sharply up to *x*/*L*_p_ = 0.03 (zone one). Zone one represents the bond anchorage length with large interfacial shear stresses. At about *x*/*L*_p_ = 0.03, the slop of the curves decreases but remains constant up to *x*/*L*_p_ = 0.3 (zone two). Within zone two, the beam is subjected to shear and bending. Finally, at about *x*/*L*_p_ = 0.3, the strain remains uniform along the CFRP plate up to the symmetry plane *x*/*L*_p_ = 0.5 (zone three). Zone three represents the constant bending moment area, where strain remains constant along the CFRP plate.

**Figure 12:**
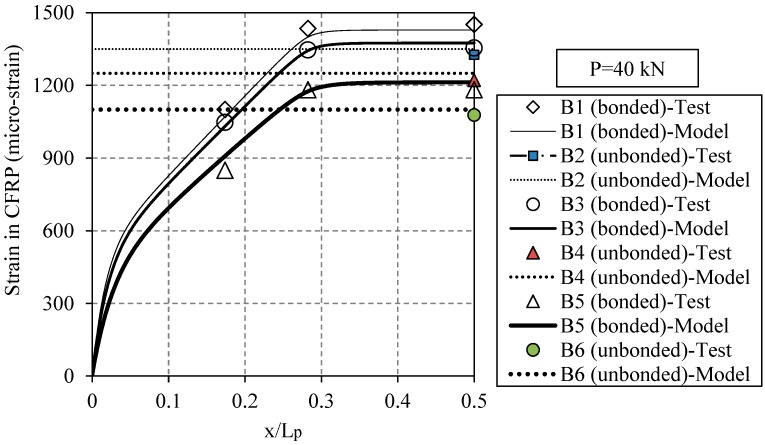
The measured and calculated strains along the bonded and un-bonded CFRP plates for the specimens strengthened with the NM, HM and UHM CFRP plates when the specimen is subjected to an actuator load level of *P* = 40 kN.

Figure 12 shows the measured and calculated strains along the bonded and un-bonded CFRP plates for the retrofitted beams with the NM, HM and UHM CFRP plates. The actuator load level is *P* = 40 kN, which is within the elastic domain. The strains were measured using the applied strain gauges. As the elastic modulus of the applied CFRP is increased, the stiffness of the plated beam also increases, which results in a smaller applied strain on the CFRP plate. The CFRP strain remains uniform along the constant bending region, which is the distance between the two external vertical loads, whereas it decreases as the bending moment decreases outside of the constant bending region. Note that the region with a high strain gradient at the plate end is associated with the anchorage length where the interfacial shear stress is at its maximum value.

### 6.3. Fatigue Test Results

In this paper, two retrofit techniques for steel members subjected to cyclic loads were discussed. The first method takes advantage of the compressive force achieved from the pre-stressed CFRP plate, and the second method is based on the reduction of stress amplitudes when the steel element is stiffened (e.g., with the UHM CFRP plates). Extensive tests were previously performed [32] demonstrating the accuracy of the first method. In this paper, the results of a series of fatigue tests will be presented to examine the effectiveness of the second retrofit technique (i.e., stiffening effect) using un-bonded CFRP plates. Therefore, in this paper, the reference un-strengthened specimen (i.e., B0) and only specimens retrofitted by the un-bonded CFRP plates are subjected to fatigue tests (i.e., specimens B2, B4 and B6). All specimens were subjected to fatigue loading between 1.7 and 18 kN.

**Figure 13:**
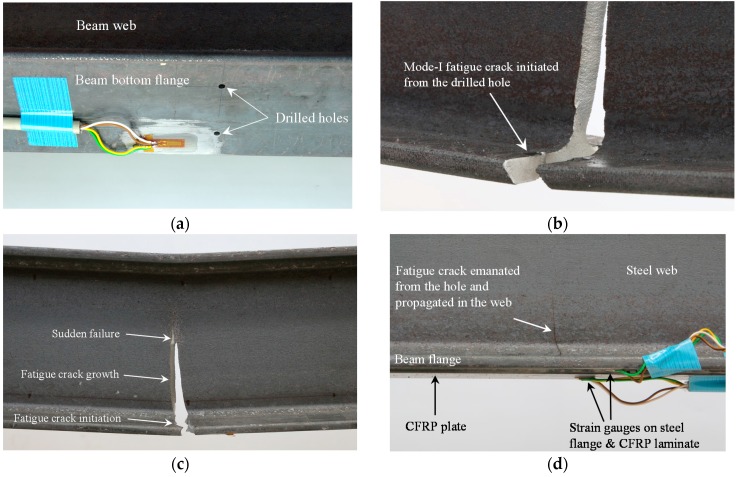
(**a**) Holes drilled in the beam bottom flange; (**b**) crack emanating from stress concentration location; (**c**) different stages of fracture failures: crack initiation, crack propagation and a sudden fracture failure; (**d**) fatigue crack initiated from the hole of the retrofitted specimen and propagated into the steel web.

The unstrengthened reference specimen, B0, was first subjected to fatigue loading. The Eddy Current NDT system was used to inspect the area close to the hole in the beam bottom flange (see Figure 13a). A fatigue crack was detected from one hole at the beam bottom flange after 450,000 cycles. After a fatigue crack was detected, the cyclic loading was continued to further study the behavior of crack propagation. The crack then continued to propagate into the beam’s bottom flange and later into the web, as shown in Figure 13b. Once the crack size reached a critical length, the overall stress intensity factor at the crack tip exceeded the fracture toughness of the steel material and the beam failed, as shown in Figure 13c.

Specimen B2 was strengthened using the un-bonded NM CFRP plate. The specimen was subjected to fatigue loading similar to the reference specimen. A fatigue crack was detected at the hole at 1,120,000 cycles. As can be observed in Figure 13d, like the reference specimen, the crack propagated in the beam bottom flange and later into the web.

Specimen B4 was strengthened using the un-bonded HM plate. It was then subjected to fatigue loading. A fatigue crack was detected from the hole after 1,120,000 cycles.

Specimen B6 was subjected to cyclic loading after it had been strengthened using the un-bonded UHM plate. This specimen survived more than 2,000,000 cycles, and no crack could be detected at the location of the holes using the eddy current NDT system as well as in a visual inspection. The number of cycles applied to each specimen and the corresponding failure mode are summarized in Table 6.

**Figure 14:**
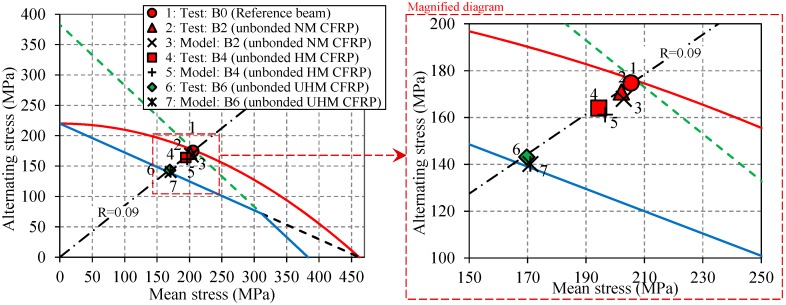
The CLD illustration of the experimental and modeling results. As the Young’s modulus of the CFRP plate increases, the stresses approach the safe zone along the original stress-ratio line.

The stresses in specimens B0, B2, B4 and B6 are presented in the CLD and shown in Figure 14. The results from the tests are compared with those obtained from the previous analytical model [38]. The red markers in Figure 14 indicate the failed specimens, whereas the green marker represents the specimens that survived after 2,000,000 cycles. In this CLD, as the stiffness of the CFRP plate increases (i.e., NM, HM and UHM, respectively), the working stresses in the CLD approach the safe region (i.e., Goodman line) along the original stress ratio line (i.e., *R* = 0.09), which verifies the hypothesis considered in Section 3. As it can be seen in Figure 14, a crack initiated from the holes in all of the specimens with working stresses far from Goodman line (i.e., B0, B2 and B4), but specimen B6, which was strengthened with the UHM CFRP laminate, survived (i.e., no crack initiation). The reason of the superior fatigue performance of specimen B6 is that its working stress is reduced substantially (due to using the UHM laminate) and is very close to the Goodman line (i.e., safe region). Nevertheless, the working stress of specimen B6 is slightly out of the safe region, which indicates that the proposed fatigue design criterion is conservative.

## 7. Conclusions

The results of the new experimental study on using a CFRP plate with a high Young’s modulus to improve the fatigue behavior of retrofitted steel beams were presented. Three different categories of the CFRP plates with the normal, high and ultra-high moduli were used for retrofitting the steel beams with the BR and UR systems. The following conclusions were drawn from the study:

1. Strengthening the steel beams using the un-bonded CFRP plates took less than half the time that was required to strengthen with the bonded CFRP plates. When strengthening with the un-bonded CFRP plates, there is no need for surface preparation or adhesive application, which can substantially reduce the time required and the cost of retrofitting.
2. By adhesively bonding the CFRP plate to the steel beams (i.e., the BR system), the relative displacements between the two components are minimized. Nevertheless, due to the elasticity of the adhesive layer, they are not exactly zero. As a result of this continuous connection, shear stress is transferred between the steel beam and the CFRP plate. In contrast, the UR system does not allow this stress transfer along the connection because there is no adhesive connection. Therefore, the BR and the UR systems produce differing behavior when shear stresses appear. Such shear stresses are only present if a shear force is acting on the system (e.g., in regions where the bending moment is not constant).
3. Two mechanisms that can shift the detail from the risky finite-life regime to the safe infinite-life regime were explained. Mechanism 1: by applying a pre-stress force to an existing fatigue-susceptible detail, the mean stress level and the stress ratio, *R*, will be reduced such that the detail is shifted from the finite-life regime to the infinite-life regime. Mechanism 2: by increasing the stiffness of the beam (e.g., in using the UHM CFRP plates), the stress ratio, *R*, is preserved, but the mean and alternating stresses will be reduced such that the detail is shifted from the finite-life regime to the infinite-life regime.
4. It was shown for the first time that the non-prestressed un-bonded UHM CFRP plates are efficient in preventing fatigue crack initiation in steel members. It is possible to use the presented retrofit approach to determine the CFRP Young’s modulus and the CFRP pre-stress to prevent future fatigue crack initiation.

## Figures and Tables

**Table 1:** Measured mechanical properties of the steel.

Steel grade	Yield strength	Tensile strength	Young’s modulus
	*f*_y,s_ (MPa)	*f*_u,s_ (MPa)	*E*_s_ (MPa)
S235JR	383	462	199,300

**Table 2:** Geometrical properties of the CFRP plates based on the manufacturers’ data sheet.

Type	Width	Thickness	Cross-sectional area
	*b*_CFRP_ (mm)	*t*_CFRP_ (mm)	*A*_CFRP_ (mm^2^)
NM CFRP	50	1.4	70
HM CFRP	50	1.4	70
UHM CFRP	50	1.4	70

**Table 3:** Mechanical properties of the CFRP plates (from the manufacturers’ data sheet).

Type	Tensile strength		Ultimate tensile strain		Young’s modulus	
	*f*_u,CFRP_ (MPa)		ε_u,CFRP_ (%)		*E*_CFRP_ (MPa)	
	Mean	Min.	Mean	Min.	Mean	Min.
NM CFRP	–	2800	–	1.70	–	165,000
HM CFRP	–	2800	–	1.35	–	205,000
UHM CFRP	1500	1200	0.34	0.27	460,000	440,000

**Table 4:** Mechanical properties of adhesive at 23 °C according to ISO 527 (from manufacturer’s data sheet).

Type	Lap shear strength (Steel to Steel)	Lap shear strength (CFRP to CFRP)
	τ_u_ (MPa)	τ_u_ (MPa)
Araldite^®^ AW 106 with Hardener HV 953 U	25	19

**Table 5:** Static test matrix.

Specimen	CFRP type	System type
B0	–	–
B1	Normal modulus	Bonded
B2	Normal modulus	Un-bonded
B3	High modulus	Bonded
B4	High modulus	Un-bonded
B5	Ultra-high modulus	Bonded
B6	Ultra-high modulus	Un-bonded

**Table 6:** Fatigue test matrix and results.

Specimen	Strengthening scheme	Laminate type	No. of cycles to crack initiation	Failure mode
B0	Unstrengthened	–	450,000	Crack initiation
B2	Un-bonded	NM CFRP	1,120,000	Crack initiation
B4	Un-bonded	HM CFRP	1,500,000	Crack initiation
B6	Un-bonded	UHM CFRP	2,000,000	Run-out

## Abbreviations

BR	Bonded retrofit
PBR	Pre-stressed bonded retrofit
UR	Un-bonded retrofit
PUR	Pre-stressed un-bonded retrofit
TPUR	Trapezoidal PUR
TriPUR	Triangular PUR
FPUR	Flat PUR
CPUR	Contact PUR
FCG	Fatigue crack growth
SIF	Stress intensity factor
CLD	Constant life diagram
NM	Normal modulus
HM	High modulus
UHM	Ultra-high modulus
CFRP	Carbon-fiber-reinforced polymer
LVDT	Linear variable differential transducer
NDT	Non-destructive testing
R	Stress ratio
D	Overall accumulated damage
FE	Finite element
